# Supplementary material for: Are carbon emissions trading and green financial instruments synergistic? -Comprehensive quantitative research based on content analysis
Source: PLoS One. 2024 Mar 7;19(3):e0298601. doi: 10.1371/journal.pone.0298601 (PMC10919601; doi:10.1371/journal.pone.0298601)
Supplement: S1 Data — (ZIP) [file pone.0298601.s001.zip › Data/地级市Z_F工作报告.zip!/╔╜╬≈/╜·│╟╩╨/└┤╫╘╣1⁄2╓┌║┼í╛┬φ┐╦╩2╛▌═°í┐_2015.docx]

各位代表：

现在，我代表市人民政府向大会报告工作，请予审议，并请市政协委员和列席人员提出意见。

一、2014年工作回顾

过去的一年，是山西不寻常的一年。面对复杂严峻的经济形势，我们深入学习党的十八大、十八届三中、四中全会和习近平总书记系列重要讲话精神，认真贯彻落实省委、省政府工作部署，坚持“稳中求进、改革创新”总基调，坚持“一争三快两率先”总战略，积极适应经济新常态，积极应对经济下行压力，认真履职，积极作为，经济社会发展稳中有进。全市生产总值完成1035.8亿元，同比增长4.7%；规模以上工业增加值增长5%；固定资产投资完成974.8亿元，增长16.4%；公共财政预算收入完成98亿元，增长3.7%；社会消费品零售总额完成332.8亿元，增长12%；外贸进出口总额完成11亿美元，增长19.5%；城镇居民人均可支配收入完成24907元，增长7.7%；农村居民人均可支配收入完成10087元，增长10.4%。

一年来，我们认真开展“转型综改攻坚年”活动。“1+9”综改试点稳步推进，下放市县两级10个部门54项行政审批权限。“气化晋城”步伐加快，中心城市规划六区管道燃气普及率达到90%。编制《晋城市低碳发展规划》，制定了“六个一”低碳示范标准。深化政府职能转变，新一轮政府机构改革基本完成。出台了行政审批改革创新的实施意见和“两集中、四到位”改革方案,取消下放行政审批项目59项，取消调整审批事项70项。煤焦公路销售体制改革全面完成，运销票据和检查站点全部取消。工商、质检、文化、城建执法等管理体制和财税、金融等重点领域改革取得新进展。山西兰花保税物流中心、晋城海关正式获批成立。晋商银行、银河证券入驻我市，城区、泽州、阳城农信社成功改制。太焦高铁有了实质性进展，今年有望开工。全面审计市属国资国企。公开发行城投债券14亿元，票面利率4.99%，为近三年全省地方债券发行招标的最低利率。组团参加了投洽会、西博会、能博会等大型经贸活动，全年招商引资签约项目163个，资金到位519.3亿元。

一年来，我们千方百计稳增长、转方式、调结构、防风险。认真落实国务院和省政府政策措施。一手减负让利，一手抓资金保障，累计为煤企减负20亿元以上，积极协调为企业融资50亿元，确保了晋城工业经济的基本面。扎实开展“项目见效年”活动，全市固定资产投资974.8亿元，超额完成“六位一体”年度目标。省市重点工程完成投资891.8亿元，增长11.5%。加快“煤、电、气、化”综合能源基地建设，推动装备制造、高新技术和文化旅游等做大做强。煤层气和电子通信增加值分别增长31.3%、33.4%，拉动规模工业增长1.6个百分点和1个百分点。出台了减轻企业负担、支持实体经济、促进就业创业、稳定金融发展的意见和加快旅游、养老、健康服务业等一系列办法措施。持续加大对实体经济、民营经济和中小微企业的扶持力度，全市民营经济增加值占到地区生产总值的59%。新创办中小微企业2886户，新增个体工商户8536户。排查民间投融资机构257家，稳妥处置各类金融风险。启动沁河古堡、炎帝陵、程灏书院规划修复工程。全市旅游总收入242亿元，增长22%。服务业增加值占到地区生产总值的37%。

一年来，我们加快新型城镇化建设。中心城市以“一园两环、三纵三横”为重点，实施了城市道路、管网、生态园林及污水垃圾处理等41项基础设施建设，新增集中供热面积160万平米。统筹推进大县城、小城镇、六大产业片区和8个“百镇建设示范镇”建设，城镇化率达到57.2%。启动建设396平方公里的环城生态圈。扎实开展改善环境质量“十组拳”攻坚行动和改善环境空气质量“百日攻坚”专项行动，一化和北岩煤矿关停，中石化油库完成选址，四个县城污水处理厂提标升级，生活垃圾无害化处理场全部建成，淘汰黄标车和老旧车辆9385辆。市区环境空气质量二级以上天数达到209天，较去年增加了67天；PM2.5、PM10浓度和空气质量综合指数分别下降28.1%、16%和19.4%，均超额完成省定指标。晋城荣获“山西省节水型城市”称号。北留镇、润城镇被命名为“国家级园林城镇”。

一年来，“三农”工作扎实推进。持续落实各项惠农政策，粮食生产虽遭受了严重自然灾害，但总产量仍然达到14.6亿斤。新建现代农业示范园区39家，新建省、市两级“一村一品”专业村286个。新发展农民专业合作社646家，总数达到4734家，创建国家级示范社12家。全年完成农产品加工销售收入63.3亿元。新农村10个连片示范区和300个新农村实现提档升级，改善农村人居环境“四大工程”扎实推进。农村土地承包经营权确权登记颁证试点工作，在16个省定试点村和2个试点乡顺利开展。培训新型职业农民1万名。农业机械化率达到68.8%，植树造林9.27万亩。任庄水库、磨河水库等水利工程进展顺利，全市自来水普及率达到92.7%。深入实施“百企千村产业扶贫”和干部下乡住村帮扶活动，对160个重点扶持村开展产业扶贫。农村居民人均可支配收入突破万元。

一年来，保障民生更加有力。公共财政用于民生支出142.7亿元，占公共预算支出的88.8%。市区开工建设中小学6所，新（改、扩）建农村幼儿园和标准化幼儿园28所。提高调整乡村医生和教师工资待遇。城区、高平、阳城通过国家义务教育均衡发展评估验收。人均基本公共卫生服务经费补助标准提高到40元。城乡居民最低生活保障标准分别增加35元和30元。全年城镇新增就业岗位4.3万人，城镇登记失业率为1.27%。城镇职工基本医疗保险报销比例，最低92%，最高97%，年最高报销额达到60万元；城镇居民基本医疗保险报销比例，最低68%，最高85%，年最高报销额达到50万元，全省最高。康复救助贫困残疾人3700名，建立了一级重度残疾人护理和生活补贴制度。48项涉农补贴资金统一“一卡通”发放。新开工建设各类保障性住房15528套，配售3168套。煤层气抽采、高硫煤深加工转化分别列入国家和省级重大科技攻关项目。图书馆、档案馆、美术馆、博物馆正式开放。城乡就业、物价涨幅等重要民生指标实现预期目标，市定十件实事全部完成。统计、地震、气象、人防、治超、民族宗教、妇女儿童、卫生计生、档案史志、外事侨务、老龄、关工委、残疾人、红十字、慈善救助等社会事业，都取得了新的成绩。

一年来，社会治理总体稳定。深入实施精神文明和民主法治建设。大力开展群众性精神文明建设，努力培育和践行社会主义核心价值观。自觉接受人大法律监督和工作监督，主动接受政协民主监督和社会监督，认真办理人大代表建议和政协委员提案627件。扎实推进“六五”普法和依法行政，社会治安综合治理不断加强，群众安全感和满意度民意测评连续三年名列全省第一。加强全民国防教育，国防后备力量建设不断提升。认真开展党的群众路线教育实践和“学习讨论落实”活动，严格执行中央“八项规定”和国务院“约法三章”，坚决反对“四风”，着力解决人民群众反映强烈的突出问题，市级“三公”经费同比下降63.5%。查处和通报了一批违法违纪案件。开展安全生产大排查大整治专项行动，制定实施安全生产责任清单，引深“知责、履责、尽责、问责”活动，安全生产形势总体平稳。全年各类安全事故下降7.9%，事故死亡人数下降1.7%。

各位代表，一年来，我们同舟共济，坚韧前行，成绩来之不易。这是我们认真贯彻执行党中央、国务院和省委、省政府一系列方针政策的结果，是市委统揽全局、正确领导的结果，是市人大、市政协有效监督、全力支持的结果，是全市人民辛勤工作、共同努力的结果。在此，我代表市人民政府，向全市人民，向驻市解放军指战员、武警官兵和公安干警，向各民主党派、各人民团体和各界人士，向各位老领导、老同志，向所有关心、支持晋城发展的同志们、朋友们，表示衷心的感谢和崇高的敬意！

同时，我们清醒地认识到：我市经济社会发展中存在的矛盾和问题仍然十分突出。煤炭行业产能过剩、价格下跌，经济运行不稳定、不确定因素增多，经济下行压力依然较大；经济总量不大、质量不优，产业结构单一，新兴产业成长不快，转型升级的任务依然艰巨；改革开放、民营经济、科技驱动、金融市场等滞后，经济增长内生动力不足，制约我市发展的一些体制机制性弊端依然存在；“三期叠加”的发展阶段，各种社会矛盾风险和安全生产任务依然十分繁重。还有：依法行政的理念还没有真正确立，行政审批环节多、时间长、效率低；一些促改革、稳增长的政策措施落实不到位，“最后一公里”的问题还没有很好地解决；一些政府机关和工作部门“四风”问题尚未根绝，工作中的形式主义、官僚主义以及缺乏担当、为官不为、懒政怠政的现象不同程度地仍然存在。

在这里，我还要特别报告：去年全市地区生产总值、财政收入等主要经济指标没有完成预期目标。一些重点工作、重点工程推进缓慢，没有实现既定进度。客观上是大环境、大形势和我们资源型经济特征共同影响的结果，也有省直有关厅局案件、土地政策变化、房地产市场低迷等因素的影响。但主要是我们对经济发展新常态的思想认识不充分、应对措施不给力，工作没有做实做好。还有“3·1”道路交通危化品燃爆特别重大事故，是建国以来第一起隧道特别重大事故，也是晋城市建市以来第一起特别重大事故。事故使人民群众生命财产遭受了巨大损失，也给山西和晋城的形象造成了损害，教训非常惨痛，十分深刻。

新的一年，形势更加严峻，任务更加艰巨。我们必须认真总结经验教训，切实增强使命意识、责任意识、忧患意识，自觉认识经济新常态，积极适应经济新常态，主动引领经济新常态，担起担子，勇于作为，全面实施“六大发展”，努力做好经济社会发展各项工作。

二、2015年工作安排

2015年，是全面深化改革的关键之年，是全面推进依法治国的启动之年，是全面完成“十二五”规划的收官之年，是我省全面推进“六大发展”的开局之年，也是晋城建市三十周年。今年政府工作的指导思想是：深入贯彻党的十八大和十八届三中、四中全会精神，以习近平总书记系列重要讲话精神为根本指针，坚持“稳中求进、改革创新”总基调，坚持“一争三快两率先”总战略，坚持“只争朝夕、一抓到底”工作要求，主动适应经济发展新常态，以提高经济发展质量和效益为中心，把转方式调结构放到更加重要位置，以转型综改试验区建设为旗帜，聚焦经济发展，深化改革开放，突出创新驱动，优化发展环境，着力民生改善，强化风险防控，推进依法行政，实现经济稳中有为，社会和谐稳定。

今年我市经济社会发展的主要目标是：地区生产总值增长6.2%左右，规模以上工业增加值增长7.5%，固定资产投资增长16%，公共财政预算收入增长6.2%，城镇居民人均可支配收入增长8%，农村居民人均可支配收入增长8%以上，城镇新增就业岗位3万个，城镇登记失业率控制在4.2%以内，居民消费价格涨幅控制在3%左右。节能减排等约束性指标，要确保完成省下达的年度任务和“十二五”目标任务。

实现上述目标，要重点做好八方面工作：

（一）改革开放

今年是全面深化改革的关键之年。我们要以“早改早主动、晚改就被动、不改要出局”的认识和态度，解放思想、创新举措，运用新思维、敢啃硬骨头，切实增强改革开放的主动性和创造性，把着力点聚焦到全市发展的重大问题上来，重点加以推进。

以综改试验区建设为旗帜和统领。不折不扣地完成省里确定的综改目标任务。按照我市三年《综改实施方案》和2015年行动计划确定的任务书、路线图和时间表，加快“1+9”综改试点和重大改革、重大事项、重大项目、重大课题推进步伐，有计划、有步骤地推进低碳城市、气化晋城、城乡统筹、循环经济、竞逐中原等各项综改工作。

推动煤炭革命。认真贯彻落实习近平总书记“能源革命”和省委“革命兴煤”、“六型”转变要求，实现煤炭产业由量的扩张到质的提升。加快煤炭主体企业改革步伐，全面深化煤炭管理体制改革，规范建立现代企业制度，完善法人治理结构，提高经营管理水平和盈利能力，切实增强企业的活力和市场竞争力。积极引导支持煤炭企业与化工、电力等行业的开放与合作，推动煤电一体、煤化联盟等，提高煤炭清洁利用和就地转化率。

进一步简政放权。严格按照机构改革方案要求，加快政府职能转变，全面完成市县两级政府机构改革。积极推进政府事权规范化、法制化，市政府今年要制定并公布权力清单、责任清单。认真做好省政府下放审批事项的承接工作，继续取消和下放市级行政审批项目。清理规范非行政许可事项和中介服务执业行为。全力打造“行政审批（许可）、公共资源交易、便民服务、效能监察”四位一体的综合性政务服务平台，全面推进行政审批“两集中、四到位”。

深化国资国企改革。以管资本增活力为方向，按照出资人政策的制定、执行、监督三分开原则，改革完善国有资产管理体制。强化国有资本经营预算，成立国有资本投资运营公司，推行信息公开，实行分类改革，实现分类监管。已实行公司制改革的国有独资、国有控股企业，要进一步理顺与出资人的关系，完善董事会制度，实行规范的法人治理；一般竞争性领域的国有企业，继续推进国有资本有序退出、合理流动，完成产权、身份双置换；公共服务类国有企业，要以政企分开、政资分开、特许经营、政府监管为主要内容，尽快建立完善现代企业制度，在加强监管的条件下放宽准入，不断提高公共资源配置效率和服务水平。同时，要统筹推进投资体制、财税金融、不动产登记和农村改革等。各县（市、区）在全面完成省市确定的各项改革任务的同时，自选动作，重点推进一至两项改革。

扩大对外开放。坚持招商引资和招才引智并重，对外开放和对内搞活并重，加快晋城海关、商检大楼建设，确保兰花保税物流中心10月封关运营，打造开放平台，发展外向型经济，努力实现对外开放新突破。加快推进太焦高铁、陵川至鹤壁、阳城至济源高速公路建设，尽快实现晋城人民“高铁梦”，尽快形成东南西北四通八达的高速公路交通体系。加强与中原经济区的经济协作，打通我市钢铁、铸管、铸件、煤机等融入郑州、洛阳等装备制造产业链的途径和渠道，实现我市与周边城市在能源供应、产品供需方面的产业互动。提升旅游业对外开放合作水平，实现资源共享、互利共赢。

（二）加快产业转型升级

集中力量转方式、调结构、提质量、增效益，把新常态下发展的基础夯实，是我市转型发展必须迈过去的一道坎。我们要保持战略定力和“功成不必在我”的韧劲，蹄疾而步稳，真正把经济工作的重心放到质量和效益上来。

做好煤炭这篇大文章。推进煤炭产业“六型”转变，巩固好晋城经济基本面。要在“保安全、保产量、保市场、保资金、促建设”上下功夫，加强煤炭市场运行监测，创新煤炭经营方式，努力开拓煤炭市场。鼓励帮助煤炭企业用好用足各种融资手段，千方百计保障煤炭企业资金链。要围绕国家新型综合能源基地建设，坚持“煤、电、气、化”多元发展，实现煤炭单一优势向综合优势转变。大力发展煤商贸、煤物流、煤转化、煤金融、煤制造等特色关联产业，建设煤炭产业新体系。

打造晋城工业经济升级版。以煤基新材料、煤基新能源为方向，科学有序发展煤化工产业，加快晋煤华昱高硫煤洁净利用化电热一体化项目、天泽乙二醇和兰花钠米碳酸钙、己内酰胺等重点项目建设。积极开发精细化、差异化的高端化工产品，实现由化肥单打到肥化并举。通过技术改造，提升清洁燃煤发电，加快阳电三期前期工作，建设千万千瓦级煤电基地。依托冶炼铸造优势，加快产业集聚、技术研发、产品集散三个平台建设，重组泽州、高平铸造企业，形成铸造—机加工—装备制造产业链条，推动晋城铸造向晋城制造飞跃。以建筑陶瓷、日用陶瓷、工艺美术瓷和新型陶瓷材料生产为核心，延伸陶瓷机械及配件、陶土采掘等陶瓷产业链条，打造陶瓷产业集群。以改革创新和科技创新为动力，激活丝麻产业，形成丝麻融合发展产业基地。同时要加强主导产业、重点企业经济运行的分析研判，及时出台针对性的办法措施，下决心清理规范取缔各类涉企收费，优化市场环境，支持实体经济、混合经济、民营经济和中小微企业共同繁荣健康发展。

加快培育新的经济增长点。发挥煤层气资源优势，以沁水煤层气总部基地建设为统领，加快煤层气集输总站、国家煤层气质检中心、煤层气交易中心、煤层气远程智能化排采系统、应急调峰设施群和煤层气综合利用等重点项目建设，努力打造我市新能源战略新优势。捆绑煤层气装备制造和气化晋城等，力争煤层气产业成为省级综改示范项目。加快推动富士康、金鼎煤机、江淮重工、力宇燃气、中道能源等向中高端延伸迈进，带动和促进我市装备制造业集群化规模化发展。落实服务业发展优惠政策，完善第三产业市场物流网点建设，积极培育一批市级重点商贸企业，统筹现代物流、信息服务、电子商务等生产性服务业和文化旅游、健康养老等生活性服务业快速发展。适应新一代科技革命，促进大数据、物联网等与传统产业、现代制造业等相结合，不断催生新兴业态。推进区域合作，打造精品线路，塑造旅游品牌，推动旅游业由观光为主向观光、休闲、度假并重转变。整合做强南太行旅游资源，叫响“太行山”品牌。完善沁河流域古堡群和古村落保护开发利用规划。要以人的城镇化为核心，在新型城镇化建设过程中发现和培育新的增长点。

全力以赴在科技创新、金融服务、民营经济发展三个突出问题上取得突破。（1）科技创新。强化企业技术创新主体地位，提升企业自主创新能力，推动高新技术产业发展，促进高新技术企业销售额与规模以上工业企业销售额快速增长。围绕支柱产业和新兴产业，积极对接国家级科研院所，加大产学研合作力度，重点在煤炭绿色开采、煤炭清洁利用、煤层气、现代煤化工、低碳技术等领域突破一批核心关键技术，形成产业发展的制高点和主动权。加快晋城市科技研发基地（科技企业孵化器）建设。（2）金融服务业。深刻认识金融对于经济发展的极端重要性，解放思想，创新实践各类金融产品和融资模式。既要发挥好政府资金引导作用，更要广泛激发社会资本活力，为市场主体源源不断地注入发展动力，不断增强金融服务实体经济的能力和水平。加快农信社改制和企业上市步伐。（3）民营经济。认真落实促进民营经济发展的各项政策和措施，坚决破除观念性、体制性、环节性障碍，放宽市场准入，消除各种隐性壁垒，帮助民营企业解决创办、创新、税费、融资、审批等方面的实际问题，让民营经济依法平等使用生产要素、公平参与市场竞争、同等受到法律保护，努力形成全民创业、万众创新的生动局面。

把投资作为稳增长的强大引擎和调结构的重要抓手。准确把握国家产业政策和投资方向，深度对接中央和全省已推出的重大工程和即将推出的新的工程包。继续坚持“六位一体”推进重点工程建设。重点实施太焦高铁、高速公路、气化晋城、环城森林生态圈、西北片区改造、金村低碳示范区、沁水煤层气总部基地、城市基础设施建设、新型城镇化、水利建设、北留—周村循环经济工业园区等一批骨干工程。一要加大招商引资力度。发挥好能源、资源、区位、产业等优势，强化产业链招商和以商招商，大力引进关联度高、竞争力强和成长性好的项目，加大招商引资与产业升级的对接力度。二要改革投融资方式方法，积极推广PPP建设模式等，在智慧城市、新能源汽车配套设施、产业园区建设、公共服务项目中，采取特许经营、购买服务、股权合作等合作方式，促进投资主体多元化，破解资金难题。三要创新用地新机制。继续推进城乡建设用地增减挂钩试点、矿业存量土地整合利用、工矿废弃地复垦利用、批而未用计划指标调剂使用，以及闲置和低效利用土地二次开发等用地新机制，多管齐下，盘活存量，用好用足各项用地政策。四要扎实推进工业园区建设。金匠新兴产业工业园、北留周村煤电化工业园、南村铸造工业园区等产业园区，要按照循环经济模式，“五规合一”，科学规划布局，形成园区循环经济发展新模式。加快基础设施建设，降低园区运营成本，吸引投资落地。健全园区管理服务机构，改革创新管理模式，完善服务体制机制，用高质量的服务争取项目落地。

（三）持续改善生态环境

牢固树立生态文明、绿色发展、低碳发展、循环发展理念，持之以恒地推进环境保护和生态建设，建设美丽晋城。

节能减排。扎实推进低碳城市“六个一”示范工作，管理机制上要开展碳排查、引入碳评价、建立碳平台、实施碳考核。严格淘汰落后产能，严控“两高”行业新增产能，抓好煤炭、冶铸、建材、化工等传统产业节能工作。以雾霾治理为重点，整治违法排污企业行为，推进工业、建筑、交通等领域节能降耗。严守生态红线和开发底线，不断提高碳汇蓄积量。

发展循环经济。抓好全产业循环链发展，大力构建资源节约型、清洁生产型、生态环保型的现代循环经济绿色产业体系。重点抓好11个省级试点、6条循环经济产业链和工业固体废物综合利用工作，加快创建“国家级循环经济示范市”。以建设国家新能源汽车推广应用示范城市为契机，加快新能源汽车推广步伐。

抓好“三化”建设。“气化晋城”。认真落实《气化晋城实施方案》，加快煤层气高压集输系统建设，实现6县（市、区）、重点工业用户、重点园区、重点乡镇等四个“全覆盖”。“绿化晋城”。主城区今年新增公园、游园面积900亩，提升改造公园面积3460亩。加快396平方公里的环城森林生态圈建设，力争2020年森林覆盖率达到45%以上。“净化晋城”。加快“井”字型骨干大水网、城乡一体化饮水安全网、农田水利灌溉网三大水网建设，开展沁河、丹河生态修复综合治理，打造花园头河、龙门湿地公园等城市生态景观。突出“控煤、治污、管车、降尘”四个关键环节，持续实施好改善环境质量“十组拳”攻坚行动。

（四）扎实推进新型城镇化

目前我市正处于城镇化快速发展期。要坚持“以人为本、优化布局、生态文明、传承文化”的基本原则，突出重点，统筹推进，切实提高新型城镇化质量和水平。

加大中心城市建设力度。以花园城市、智慧城市和文明城市为目标，围绕“拓展、改造、提质”，强化中心城市建设，以崭新的城市形象迎接建市30周年。按照“六区联动、组团发展”的城市空间总体布局和“一园两环、三纵三横”的城市建设主框架，加快文博路、景西路、书院街、207国道改线等续建工程建设，畅通拥堵点，促进大循环，形成快速高效、级次匹配的完整路网体系和功能完善的公共服务基础设施布局体系。今年重点实施泽州路、太行路、文昌街、新市东街等7条城市道路改造提升工程。打通主城区和片区连接线，加快金村片区、西北片区、兰花路片区、北石店片区等的改造扩容速度，推动六区互动联动。统筹推进山水园林路、“三供两处理”、保障性住房、城中村改造，建设布局合理、功能完善、生活方便、生态良好、管理创优的城市新形象。

抓好大县城和产业片区建设。围绕全省“一核一圈三群”城镇化布局，结合我市“一城两翼”城镇化架构，“四位一体”推进城镇化建设。实施“大县城”战略，积极引导生产要素、优势资源向大县城集中，加快扩容提质步伐，增强县城对县域经济的带动作用和吸纳集聚人口的能力。突出产业片区主阵地作用，以工业化推动城镇化，加快道路、水、电、暖、气等基础设施建设，完善配套就业、教育、住房、医疗、文化等综合公共服务，兴业聚人扩镇，集中打造巴公、南村、马村、北留-周村、嘉峰、米山等各具特点的产业片区，推动农民就地城镇化。把采煤沉陷区治理、农村危房改造、地质灾害治理、易地扶贫搬迁等纳入城镇化建设的总体规划，与产业开发、就业创业、农民致富、土地整治、生态修复、人居环境结合起来整体推进。

提升城市管理水平。强化城市规划的实施管理，维护城市规划的权威性和严肃性。积极引导社会力量和公民企业参与城市管理，做到政府监控、市场调节、公民参与三管齐下，实现政府、市场、社会的良性互动。坚持建管并重、标本兼治，集中开展“道路提升、环境整治、交通畅通”三项治理，重点对环境卫生、交通拥堵、违章建筑、无序设摊、跨门营业等顽症集中整治。解决好脏、乱、差、堵等人民群众关心的突出问题。积极利用现代信息技术，推进“智慧城市”建设。

（五）进一步做好“三农”工作

坚持把“三农”工作作为重中之重，以土地制度改革为重点，促进农民稳定增收、农业产业发展和农村环境改善。

以土地制度改革为重点，深化农村改革。全面开展土地承包经营权确权登记颁证工作，探索建立流转农业用地风险保障金制度，引导土地经营权有序流转。在泽州县率先开展农村集体经营性建设用地入市试点，通过出让、租赁、入股和抵押等方式入市流转。对农村宅基地进行确权登记颁证，在保障农户宅基地用益物权前提下，积极探索推进农民住房财产权抵押、担保、转让，扩展宅基地使用权。开展农村集体经济组织产权改革试点，赋予农民对集体资产股份占有、收益、有偿退出及抵押、担保、继承权。

以特色产业为重点，促进现代农业发展。在稳定粮食生产的基础上，大力发展畜牧、蔬菜、蚕桑、食用菌、干鲜果、小杂粮、中药材、苗木花卉等特色现代农业产业，加快农业产业园区建设。发挥农业产业化龙头企业作用，整合优势品牌，密切与农户、农民合作社的利益联结关系，促进农村一二三产融合发展，走集群化、产业化道路。引导农村土地承包经营权向种植能手、农民专业合作社、家庭农场、专业大户、农机专业服务组织等新型经营主体流转，鼓励发展多种形式适度规模经营。培训新型职业农民8000人。

以农村人居环境改善为重点，加强新农村建设。继续实施好完善提质、农民安居、环境整治、宜居示范“四大工程”，重点抓好美丽宜居示范村“百村”建设，完成2715户农村危房改造，开工建设30个村的生活污水处理工程，启动31个村4426户12211人的采煤沉陷区搬迁安置。创新扶贫开发机制，推进精准扶贫。继续实施产业扶贫、百企千村产业扶贫工程和领导干部下乡住村、市县单位结对帮扶、党员干部帮贫到户工作。抓好易地移民搬迁工作，完成移民搬迁5000人。

（六）着力保障和改善民生

坚持守住底线、突出重点、完善制度，着力构建普惠、均衡、优质的公共服务体系，确保我市社会福利水平位居全省前列。

优先发展教育，为晋城未来发展提供源源不断的人才支撑。积极推进义务教育均衡发展，强化公办学校尤其是农村义务教育薄弱学校标准化建设的投入机制和长效机制，扩大城乡教师岗位交流，让每一个孩子都能接受公平的有质量的教育。提升特殊教育，抓好学前教育、民办教育、社区教育和继续教育，构建全民终身教育体系。新改扩建标准化幼儿园15所，加快市区续建中小学速度，加快发展现代职业教育。加快金村教育园区建设。

提升医疗卫生计生服务水平。继续深化医药卫生体制改革，积极预防重大传染病发生，健全重特大疾病医疗保险和救助制度，提高重大突发公共卫生事件处置能力。完善城乡居民大病保险制度，加快发展商业健康保险。进一步完善基本药物制度，做好常用低价药品供应保障工作。落实乡村医生多渠道补偿政策，鼓励和引导社会资本开办医疗机构。落实好计生政策，促进人口长期均衡发展。积极抓好分级诊疗，进一步健全完善医疗服务体系，建设健康晋城。

做好创业就业工作。认真落实市政府关于进一步加强创业就业工作的26条措施，改善创业环境，开展创业培训，拓展就业渠道，鼓励高校毕业生、失业人员、返乡农民工大胆创业，以创业带动就业。加快发展服务业等劳动密集型企业，充分发挥中小微企业就业主渠道作用。

加强社会保障工作。认真落实城乡居民基本养老保险制度实施意见，提高城乡低保标准，扩大养老、医疗保险覆盖范围,认真做好全国社会保障卡综合应用试点建设工作，加快各类养老基础设施建设。结合机关事业单位养老保险制度改革，建立工资正常调整和增长机制。降低流通环节费用，搞好粮、油、肉、蛋、菜、糖等生活必需品的生产储备和应急商品的储备，保持物价总体平稳。

加快发展科技、文化、体育等社会事业。大力实施创新驱动和人才强市战略，充分发挥科技人才在转变经济发展方式和调整经济结构中的支撑引领作用，加大科技创新投入，加强科技队伍建设。继续实施文化强市和文化惠民工程，深化文化体制改革，加强基层基础建设，培育文化产业发展能力，加快构建现代公共文化服务体系。高度重视文物保护工作，深度挖掘历史文化内涵，弘扬我市优秀的法治文化、廉政文化、红色文化，彰显文化特色，提升文化软实力。广泛开展全民健身运动，促进群众体育和竞技体育全面发展。大力发展老龄、残疾人、慈善、红十字事业，继续做好地震、气象、人防、对台、档案史志、民族宗教、外事侨务、广播电视、妇女儿童等各项工作。

各位代表，民之所望，施政所向！我们一定把民生改善作为一切工作的出发点和落脚点，坚持优先保障民生投入、优先安排民生项目、优先解决民生问题，在经济下行压力较大的情况下也要让全市人民过上好日子！

（七）切实抓好社会治理创新

坚持系统治理、依法治理、综合治理、源头治理，提高社会治理法治化水平，促进社会长治久安。

加强民主法治建设。围绕法治晋城目标，深入开展法治宣传教育，集中抓好国家宪法日和依法行政宣传月活动。不断丰富民主形式，拓宽民主渠道，健全民主制度，推进决策、执行、管理、服务、结果全方位公开，切实保障人民群众的知情权、参与权和监督权。大力推进严格执法、公正司法、全民守法，全面提高社会治理水平。

加强精神文明建设。加强公民和未成年人思想道德建设，培育和践行社会主义核心价值观。弘扬晋城精神，深化社会公德、职业道德、家庭美德和个人品德教育。倡导全民阅读，推进文明旅游、志愿服务、诚信建设三项工作制度化、法治化。充分发挥“晋城好人”等道德模范的示范引导作用，培育良好社会风尚，塑造文明晋城新形象。继续抓好文明城市、村镇、单位创建活动。

加强和创新社会治理。健全落实领导责任制，建立立体化社会治安防控体系，推进社会治安综合治理。建立健全社会矛盾排查研判和预警机制，深入推进矛盾纠纷调解体系建设。强化对危害食品药品安全、影响安全生产、损害生态环境、破坏网络安全等重点问题的治理，妥善处理各类应急突发事件。继续加强国防动员、国防教育和国防后备力量建设，积极开展双拥工作，促进军民融合发展。

切实抓好安全生产。深刻汲取“3·1”特别重大事故教训，牢固树立安全生产“红线”意识。严格落实新修订的《安全生产法》，坚持“党政同责、一岗双责、齐抓共管”。继续开展安全生产“四责”活动，着力形成人人知责、认真履责、个个尽责、严格问责的安全生产责任制新格局。大力开展安全生产大检查和打非治违专项行动，全面加强煤矿、煤层气、道路交通、消防、非煤矿山和危化品、水库、人员密集场所以及建筑施工、油气管道、城市燃气、特种设备等各行业各领域的安全生产工作。强化应急管理和考核问责，严格落实安全生产目标责任考核“一票否决制”，按照“四不放过”原则，严肃事故查处，依法责任追究，坚决杜绝重特大事故、减少一般性事故。

（八）加强政府自身建设

适应新常态，必须在状态。做好今年各项工作，必须以扎实开展好学习讨论落实活动为契机，强化依法行政，转变政府职能，狠抓工作落实，加快建设法治、创新、廉洁和服务型政府。

坚持依法行政。认真贯彻十八届四中全会精神和省、市《实施意见》，运用法治思维和法治方式全面正确履行职能，推进机构、职能、权限、程序、责任法定化，做到“法无授权不可为、法定职责必须为”。深化行政执法体制改革，合理配置执法力量。建立重大决策公开征求意见和民主协商制度，完善政府法律顾问和智库制度，探索重大决策终身责任追究制度及责任倒查机制，保障法律的正确实施。完善新闻发布制度，全面推进政务公开。严格执行人大及其常委会的决议决定，主动接受人大、政协监督，认真办理人大代表议案、建议和政协提案。广泛听取民主党派、工商联、无党派人士意见，充分发挥工会、共青团、妇联等人民团体作用。支持法院、检察院依法独立公正行使职权。努力建设职能科学、权责法定、执法严明、公开公正、廉洁高效、守法诚信的法治政府。

狠抓工作落实。人民代表大会通过的《政府工作报告》，是人民意志的集中体现，具有法律效应。落实好《政府工作报告》中确定的各项目标任务，是各级政府各个部门的法定责任。会后，我们将细化“责任分解表”，制定“推进路线图”，以目标倒逼进度，以督查强化落实，推动政府工作提速提效。要守纪律，讲规矩，加强公务员队伍建设，强化干部问责、问绩、问效措施，坚决纠正、严肃处理各级各部门，尤其是“一把手”这个“关键少数”不敢担当、不愿担当、不会担当，做选择、打折扣、搞变通、为官不为等工作态度和行为。紧紧围绕改革发展稳定大局，全市“一盘棋”，以上率下，真抓实干，奋发有为，只争朝夕，以良好的作风确保各项工作顺利推进、落实到位。

实施“六权治本”。围绕依法确定权力、科学配置权力、制度约束权力、阳光使用权力、合力监督权力、严惩滥用权力的要求，建立形成不敢腐、不想腐、不能腐的权力运行长效机制。认真落实“两个责任”，严格执行党风廉政建设责任制和廉洁从政各项规定，坚决反对和克服“四风”。从严控制机构编制和财政供养人员，压减“三公”经费支出，推进公务用车改革。加强政府采购资金、政府投资项目、国有土地使用权出让等管理与监督，加强行政监察、项目稽查和审计监督，虚心接受社会监督和舆论监督。严肃查处各类违纪违法案件，做到干部清正、政府清廉、政治清明。

在做好以上各项工作的同时，各级政府要科学编制好“十三五”规划，谋划好发展蓝图。今年还要投入8.8亿元，办好十件实事：1、为全市36万60岁以上居民免费健康体检；2、为参加新农合农民办理市域就医便民结算一卡通；3、解决5.3万山区农村人口的饮水安全问题；4、提高农村低保标准平均每人每月20元；5、完成农村公路提质工程400公里；6、建立城乡统一的居民养老保险制度；7、建设城乡社区老年日间照料中心100个；8、启动采煤沉陷区治理工程；9、新增城市公交车辆92台；10、新增市区公共自行车3000辆、站点50个。

各位代表，改革催人奋进,发展任重道远。让我们紧密团结在以习近平同志为总书记的党中央周围，在市委的坚强领导下，同心同德，奋发有为，努力完成今年各项目标任务，为开创“弊革风清、富民强市”新局面而努力奋斗！
